# Redox-dependent dimerization of p38α mitogen-activated protein kinase with mitogen-activated protein kinase kinase 3

**DOI:** 10.1074/jbc.M117.785410

**Published:** 2017-07-24

**Authors:** Rekha Bassi, Joseph R. Burgoyne, Gian F. DeNicola, Olena Rudyk, Vittorio DeSantis, Rebecca L. Charles, Philip Eaton, Michael S. Marber

**Affiliations:** From the King's College London British Heart Foundation Centre of Excellence, Department of Cardiology, The Rayne Institute, St. Thomas' Hospital, London SE1 7EH, United Kingdom

**Keywords:** mitogen-activated protein kinase (MAPK), oxidative stress, p38, protein complex, protein kinase

## Abstract

The kinase p38α MAPK (p38α) plays a pivotal role in many biological processes. p38α is activated by canonical upstream kinases that phosphorylate the activation region. The purpose of our study was to determine whether such activation may depend on redox-sensing cysteines within p38α. p38α was activated and formed a disulfide-bound heterodimer with MAP2K3 (MKK3) in rat cardiomyocytes and isolated hearts exposed to H_2_O_2_. This disulfide heterodimer was sensitive to reduction by mercaptoethanol and was enhanced by the thioredoxin-reductase inhibitor auranofin. We predicted that Cys-119 or Cys-162 of p38α, close to the known MKK3 docking domain, were relevant for these redox characteristics. The C119S mutation decreased whereas the C162S mutation increased the dimer formation, suggesting that these two Cys residues act as vicinal thiols, consistent with C119S/C162S being incapable of sensing H_2_O_2_. Similarly, disulfide heterodimer formation was abolished in H9C2 cells expressing both MKK3 and p38α C119S/C162S and subjected to simulated ischemia and reperfusion. However, the p38α C119S/C162S mutants did not exhibit appreciable alteration in activating dual phosphorylation. In contrast, the anti-inflammatory agent 10-nitro-oleic acid (NO_2_-OA), a component of the Mediterranean diet, reduced p38α activation and covalently modified Cys-119/Cys-162, probably obstructing MKK3 access. Moreover, NO_2_-OA reduced the dephosphorylation of p38α by hematopoietic tyrosine phosphatase (HePTP). Furthermore, steric obstruction of Cys-119/Cys-162 by NO_2_-OA pretreatment in Langendorff-perfused murine hearts prevented the p38-MKK3 disulfide dimer formation and attenuated H_2_O_2_-induced contractile dysfunction. Our findings suggest that cysteine residues within p38α act as redox sensors that can dynamically regulate the association between p38 and MKK3.

## Introduction

p38 MAPK (p38) family members coordinate, expand, and transmit intricate signals from cell surface receptors and other environmental cues. Like that of their counterparts ERK and JNK, p38 activation is classically orchestrated through a three-step cascade made up of the MAPK, another upstream MAPK kinase (MAPKK/MKK/MEK), and another, further upstream MAPKK kinase (MAPKKK or MEKK) (1). All four p38 MAPK isoforms, α, β, γ, and δ, share significant sequence homology (2) but differ in their expression patterns, activation profiles, and substrates, suggesting divergent cellular functions. p38α is ubiquitously expressed and is the only isoform essential for development, because its absence results in embryonic lethality (3). Consequently, it is the most widely studied isoform, implicated in numerous biological and pathological processes.

p38α interfaces with a diverse set of partners, including upstream activators, deactivating phosphatases, and substrates. To confer fidelity and regulate the extensive input signals to the appropriate cellular outcomes, p38α, like all other MAPKs, uses a docking strategy to specifically bind to its interacting partners. Linear sequence motifs are employed within interacting partners that recognize features on p38α remote from the kinase active site. Of these, the most ubiquitous are the D-motifs (4). Crystallographic studies, together with mutagenic analyses of docking interactions of p38α, have revealed that the D-motif–binding site is formed of short peptide sequences composed of two subsites, recognizing an acidic patch (CD domain) and a hydrophobic docking groove (5, 6). Interestingly, the p38α substrate and upstream activators, MEF2a and MKK3b, respectively, were reported to interact with p38α at the D-motif–binding site (7). They both appeared to engage the hydrophobic docking groove and induced similar local but different distant conformational changes within p38α. Pertinent to this, more recent crystallographic data of p38α bound to a peptide derived from MKK3b (containing the D-motif consensus sequence) induced a third conformation, distinct from unphosphorylated and phosphorylated p38α (8). The exact mode by which a protein partner binds the common docking site on p38α can therefore have profound consequences on the structure and presumably the function of p38α.

The oxidation of protein cysteine residues has emerged as an important means of regulating protein kinase function. This form of protein regulation is elicited by an imbalanced cellular redox state due to changes in reactive oxygen or nitrogen species formation or alterations in the cellular antioxidant or reducing system (9). The redox sensitivity of a protein cysteine residue is very dependent on its precise surrounding microenvironment. Those cysteines with a low p*K_a_*, due to the presence of neighboring basic amino acids lysine, arginine, or histidine, tend to be reactive with oxidants because they are readily deprotonated. Low p*K_a_* thiols exist as a thiolate anion (S^−^) at neutral cellular pH, making them more reactive with electrophillic oxidants compared with those in the protonated thiol state (10). A reactive thiol can undergo a diverse range of oxidative modifications that is dependent on the oxidant species present. Because p38α is activated by oxidative stress, we hypothesized that this could be mediated through direct thiol oxidation. Certainly, this is consistent with the major upstream p38α kinase, MKK3, having a cysteine adjacent to its D-motif that forms an intermolecular disulfide with p38α when cocrystallized (8). Here, we investigated whether redox-sensitive cysteines in p38α MAPK influence its activation during oxidant stress.

## Results

### Oxidants induce reversible higher-molecular weight forms of p38 and MKK3

**Figure 1.**
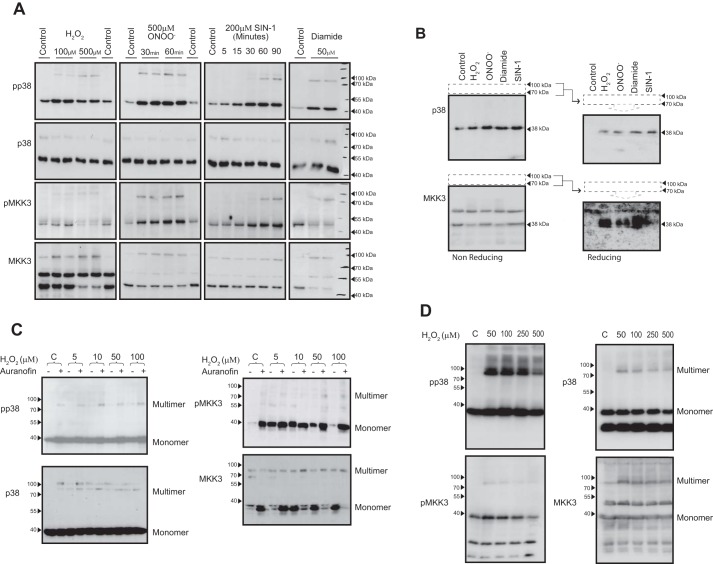
**Oxidants induce reversible higher molecular weight forms of p38 and MKK3 in rat myocardium.** *A*, non-reducing SDS-PAGE and immunoblot analysis of AVRMs incubated with H_2_O_2_, ONOO^−^, SIN-1, or diamide. *B*, SDS-PAGE and immunoblot analysis of disulfide complexes (excised from non-reducing SDS-PAGE separation as in *A*) resolved under reducing conditions (by inclusion of 2-mercaptoethanol). *C*, non-reducing SDS-PAGE and immunoblot analysis of AVRM incubated with H_2_O_2_ in the absence or presence of auranofin. The p38 and MKK3 multimers are subject to continuous cycling. *D*, non-reducing SDS-PAGE and immunoblot analysis of rat hearts exposed to H_2_O_2_ during aerobic Langendorff perfusion. H_2_O_2_ induces the formation p38 and MKK3 multimers in the intact heart in a manner similar to those seen in AVRMs in *A–C*.

Exposure of adult rat ventricular myocyte (AVRMs)^2^ to H_2_O_2_, peroxynitrite, SIN-1, or diamide, oxidants previously described as inducing activation of p38, revealed the existence of both monomeric and multimeric species of p38 by immunoblot analysis following SDS-PAGE under non-reducing conditions (Fig. 1*A*). The majority of p38 was detected as a band that resolved at the expected relative molecular mass of ∼40 kDa. We observed that p38 not only became phosphorylated following exposure to 100 μm H_2_O_2_, 500 μm ONOO^−^, 200 μm SIN-1, or 50 μm diamide, as expected, but these also induced the appearance of phospho-p38α migrating at a higher apparent molecular mass between 70 and 100 kDa (Fig. 1*A*). Analysis of these cell lysates also revealed activation of MKK3, the prototypical upstream kinase activator of p38, under identical conditions. Similar to p38, MKK3 was present in both monomeric and multimeric forms. To demonstrate that the formation of oxidant-mediated multimers was due to disulfide bond formation, the complexes were first isolated from a non-reducing SDS-PAGE using the 70 and 100 kDa molecular mass markers as a guide to excise the relevant portion of the gel, indicated by *dashed frames* (see Fig. 1*B*). The portion of the gel containing the multimeric complexes was then overlaid onto a fresh SDS-polyacrylamide gel and resolved under reducing conditions, by the inclusion of 2-mercaptoethanol (Fig. 1*B*). Subsequent immunoblot analysis detected total p38 and MKK3 as bands now resolved at ∼38 kDa and the absence of multimeric forms, verifying that complexes contained MKK3 and p38 bound by reversible disulfide bonds. The lack of p38 and MKK3 in the control lanes under reducing conditions demonstrates that they did not interact under basal conditions (non-oxidizing) and therefore did not form a higher-molecular weight complex and were absent from the portion of the gels indicated by the *dashed frame*.

### The p38 and MKK3 multimers are probably subject to continuous redox cycling

Because the oxidant-induced higher-molecular weight complexes containing p38 and MKK3 represented a very small portion of the total cellular p38/MKK3 protein, we wondered whether disulfide reductase enzymes (11), such as thioredoxin, were limiting multimer accumulation. Administration of 2 μm auranofin, a thioredoxin-reductase inhibitor, for 30 min prior to the exposure to H_2_O_2_ resulted in an accumulation of the p38- and MKK3-containing multimer (Fig. 1*C*). Auranofin was so effective in trapping the oxidized multimer that it could be detected at low concentrations and even in the absence of H_2_O_2_. These observations suggest that the p38/MKK3-containing multimers undergo continuous redox cycling within the cellular environment and can therefore be dynamically regulated by endogenous oxidant formation.

### H_2_O_2_ induces the formation p38 and MKK3 multimers in isolated rat hearts

We next examined rat hearts exposed to H_2_O_2_ during aerobic perfusion to assess whether the H_2_O_2_-induced multimers that we observed in AVRMs also occurred in the more complex and physiological environment of the intact heart. We noticed that monomeric forms of both phosphorylated p38 and MKK3 were detected in hearts that underwent baseline aerobic perfusion, and multimeric forms appeared only upon exposure to H_2_O_2_ in a dose-dependent manner (Fig. 1*D*). Monomeric bands are determined by the immunosignal that resolves at the apparent molecular mass of p38 and MKK3 (∼40 kDa), according to the antibody supplier. p38 and MKK3 exist largely in monomeric form (reduced form). A small proportion becomes complexed following exposure to H_2_O_2_. Consequently, the monomer bands are overexposed. Nonetheless, the abundance of multimer relative to monomer seemed greater than in isolated cells, reinforcing potential pathophysiological relevance and encouraging further analysis.

### The p38- and MKK3-containing multimers are heterodimers

**Figure 2.**
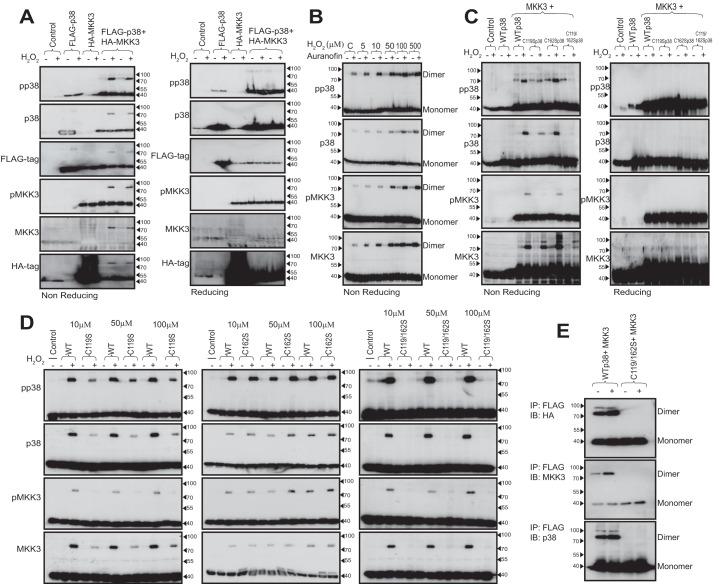
**The p38 and MKK3 containing multimers are heterodimers dependent on Cys-119 and Cys-162 of p38.** *A*, non-reducing and reducing SDS-PAGE and immunoblot analysis of HEK293 cells overexpressing FLAG-tagged WT p38α and/or HA-tagged MKK3 in the presence or absence of 100 μm H_2_O_2_. The anti-FLAG signal seen in the *HA-MKK3 lanes* is the result of “bleed-over” from neighboring lanes. *B*, non-reducing SDS-PAGE and immunoblot analysis of HEK293 cells overexpressing WT p38α and MKK3 incubated with H_2_O_2_ in the absence or presence of auranofin. The H_2_O_2_-mediated dimers are subject to continuous cycling. *C*, non-reducing and reducing SDS-PAGE and immunoblot analysis of HEK293 cells overexpressing WT p38α or p38α-cysteine mutants lacking either Cys-119, Cys-162, or both and treated with 100 μm H_2_O_2_. Cysteines 119 and 162 of p38α are both involved in disulfide dimer formation, but abundance is more dependent on Cys-119. *D*, non-reducing SDS-PAGE and immunoblot analysis of HEK293 cells overexpressing WT p38α or p38α-cysteine mutants and treatment with 10, 50, or 100 μm H_2_O_2_. MKK3 and p38α form a heterodimer dependent on Cys-119 and Cys-162. *E*, non-reducing SDS-PAGE and immunoblot analysis (*IB*) of HEK293 cells overexpressing WT p38α or C119S/C162S p38α and treated with 100 μm H_2_O_2_, following FLAG immunoprecipitation (*IP*).

Observations so far indicate that oxidant-induced, high-molecular weight, disulfide-dependent, reversible aggregates form that contain MKK3 and p38 immunoreactivity. The apparent molecular weight suggests these are either disulfide-bound hetero- or homodimers. To discern between these possibilities, we expressed FLAG-tagged WT p38α and HA-tagged MKK3 in HEK293 cells. Using both non-reducing and reducing SDS-PAGE immunoblot analysis of lysates of cells overexpressing p38α and MKK3 in the absence or presence of H_2_O_2_, we were able to largely recapitulate the observations in AVRMs (Fig. 1, *A–C*), isolated heart preparations (Fig. 1*D*), and HEK293 cells (Fig. 2*A*). In the presence of H_2_O_2_, p38α and MKK3 both became phosphorylated. Despite effective ectopic overexpression of these proteins, a higher-molecular weight complex was only seen when MKK3 and p38α were co-expressed, suggesting that disulfide-bound homodimers do not form. These observations were verified using anti-FLAG and anti-HA antibodies (Fig. 2*A*). The administration of 2 μm auranofin 30 min prior to exposure to H_2_O_2_ in cells overexpressing both p38α and MKK3 led to the accumulation of the disulfide dimer spontaneously and at low concentrations of H_2_O_2_ (Fig. 2*B*), thus reinforcing the notion that the amount of oxidized p38α that we observe is limited by the continued reductive action of endogenous thioredoxin.

### Cysteines 119 and 162 of p38α are involved in disulfide dimer formation

To further examine the formation of the H_2_O_2_-mediated heterodimer, we utilized a web-based program developed by Ricardo Sanchez and Jamil A. Momand (12) to predict which of the four cysteine residues of p38α were likely to form disulfides. We entered published structural data deposited in the Protein Data Bank (entry 1LEZ), describing the crystal structures of p38α complexed to the docking sites on its nuclear substrate MEF2a and activator MKK3b (7). The results suggested that Cys-119 and Cys-162 could be potential disulfide-forming thiols located near the surface of the protein. Furthermore, the presence of neighboring lysine and histidine residues renders them more susceptible to electrophilic attack by lowering their *pK_a_*. This, together with the observation that Cys-119 and Cys-162 may participate in hydrophobic contacts with the MAPK docking sites within MKK3b- and MEF2a-derived peptides (7), led us to substitute either or both of these residues with serine. We found that p38α-cysteine mutants lacking either Cys-119, Cys-162, or both resulted in attenuation, enhancement, or abolition, respectively, of H_2_O_2_-mediated p38α-MKK3 dimer formation (Fig. 2*C*). We believe that the formation of a reversible interdisulfide bond between p38α and MKK3 occurs preferentially as a result of oxidation of the sulfhydryl group on Cys-119 by H_2_O_2_, because mutation of this residue attenuated the formation of the dimer. In contrast, cells containing intact Cys-119 but mutated Cys-162 (C162S) have a similar or greater abundance of the dimer (Fig. 2*C*). The double mutant (C119S/C162S) did not form any detectable dimer following treatment with 100 μm H_2_O_2_. These differences in propensity to p38α-MKK3 disulfide dimer formation between the cysteine mutants were verified on multiple repetitions and at lower, more physiological, concentrations of H_2_O_2_ (see Fig. 2*D*). Furthermore, the presence and absence of dimer in immunoprecipitates from cells co-expressing FLAG-MKK3 and HA–WT p38α or C119S/C162S p38α, respectively, confirmed that MKK3 and p38α form a disulfide-bound heterodimer dependent on these residues (Fig. 2*E*). Despite the abolition of disulfide dimerization with the C119S/C162S p38α mutant, transphosphorylation of the p38α activation loop by MKK3 seems to proceed normally based on immunoreactivity of the monomeric form with anti-pTGpY dual phosphospecific antibody (Fig. 2, *C* and *D*). We therefore examined whether the ability to activate p38α was disulfide dimer–dependent in another more pathophysiologically relevant cellular model.

### Simulated ischemia and reperfusion causes p38α-MKK3 disulfide dimer formation dependent on Cys-119 and Cys-162 of p38α

**Figure 3.**
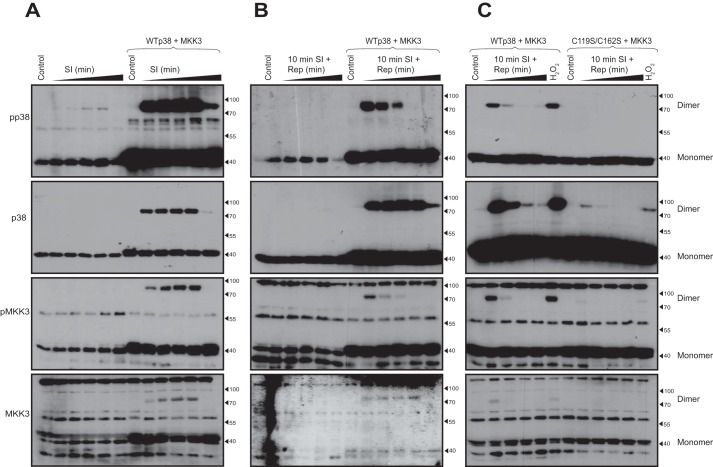
**Simulated ischemia and reperfusion causes p38α-MKK3 disulfide dimer formation dependent on Cys-119 and Cys-162 of p38α**. *A*, non-reducing SDS-PAGE and immunoblot analysis of H9C2 cells expressing endogenous or exogenous WT p38α and MKK3 subjected to 5, 10, 20, or 40 min of simulated ischemia. *B*, non-reducing SDS-PAGE and immunoblot analysis of H9C2 cells expressing endogenous or exogenous WT p38α and MKK3 subjected to 5, 10, 20, or 40 min of simulated ischemia, followed by 2.5, 5, 10, 20, or 40 min of simulated reperfusion. *C*, non-reducing SDS-PAGE and immunoblot analysis of H9C2 cells overexpressing WT p38α or C119S/C162S p38α and MKK3, subjected to 10 min of simulated ischemia and 2.5, 5, 10, 20, or 40 min of reperfusion.

We (13–15) and others (12, 16) have previously reported that p38α becomes activated during myocardial ischemia and reperfusion. We investigated the role of dimer formation employing an *in vitro* model of simulated ischemia–reperfusion in a rat myoblast cell line (H9C2) that approximates the clinical setting of acute myocardial infarction (13). Exposure of native and transfected H9C2 cells to simulated ischemia (SI) led to p38 phosphorylation and formation of the p38α-MKK3 disulfide dimer (Fig. 3*A*), mimicking the effect of H_2_O_2_. The phosphorylation of monomeric p38α and MKK3 occurred in a time-dependent manner. The disulfide dimer was detectable after 5 min of SI, continued to increase until 20 min, and diminished to baseline after 40 min of SI. These observations were replicated in cells overexpressing p38α and MKK3, albeit with a marked increase in p38α activation compared with untransfected cells. The level of disulfide dimer formed during SI was notably higher than with 500 μm H_2_O_2_. To investigate the effect of simulated reperfusion on the disulfide dimer formation, we subjected cells to 10 min of SI and increasing durations of reperfusion. As expected, disulfide dimer was detected in cells following 10 min of SI, with phosphorylation of both p38α and MKK3. The disulfide dimer was also detected in cells following all of the durations of reperfusion that we tested. The phosphorylation of p38α and MKK3 within the disulfide dimer was most marked at the 2.5- and 5-min time points of reperfusion (Fig. 3*B*). Identical experiments carried out on cells expressing the double cysteine mutant of p38α confirmed that disulfide dimers were dependent on Cys-119/162, but the presence or absence of disulfide dimers did not appreciably alter dual phosphorylation of p38α (Fig. 3*C*). Thus, in keeping with the results in HEK293 cells (see Fig. 2, *C* and *D*), abolishing disulfide dimer formation does not seem to impact p38 activation as assessed by dual phosphorylation of monomeric p38. We therefore examined further biochemical detail of the relevant cysteines to determine whether they can form adducts with small molecules that could sterically hinder recognition of MKK3 by p38α.

### Dibromobimane (dBBr) specifically reacts with closely spaced cysteine sulfhydryl groups

**Figure 4.**
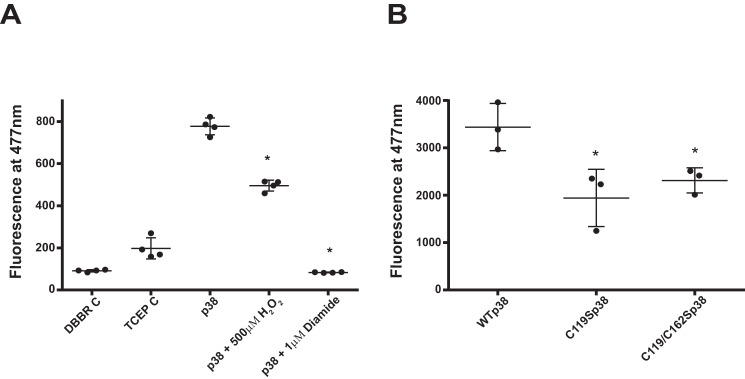
**dBBr reacts with wild-type and mutated p38α**. *A*, dBBr fluorescence measurements from three separate experiments of WT p38α incubated with dBBr, in the absence and presence of 500 μm H_2_O_2_ or 1 μm diamide. Quantification data are shown as mean ± S.E. (*error bars*). *, *p* < 0.05 *versus* p38, by one-way ANOVA and Tukey's test. *B*, dBBr fluorescence measurements from three separate experiments of WT p38α, C119 p38α, or C119S/C162S p38α incubated with dBBr. Quantification data are shown as mean ± S.D. (*error bars*). *, *p* < 0.05 *versus* WT p38α, by one-way ANOVA and Newman–Keuls test.

The divergent effects of C119S and C162S on dimer formation (Fig. 2, *C* and *D*) suggest that they could be acting as vicinal thiols that are asymmetrically coupled. To determine the distance between Cys-119 and Cys-162, we exposed WT p38α to dBBr, which emits at 477 nm when both of its alkylating groups, separated by 3–6 Å, become covalently linked to available reactive thiols. Incubation of WT p38α with dBBr alone resulted in a significant increase in fluorescence, compared with the dBBr and tris(2-carboxyethyl)phosphine (TCEP) controls (Fig. 4*A*). The inclusion of 500 μm H_2_O_2_ led to a marked reduction of fluorescence, indicating that redox-sensitive cysteines had been oxidized and were unable to react with the dBBr. Preincubation of p38α with diamide completely abolished the fluorescence signal with dBBr, confirming that the signals observed were a result of dBBr-specifically cross-linking vicinal thiols within p38α.

In an attempt to verify our earlier observation that Cys-119 is the culpable thiol involved in the formation of an interdisulfide bond between p38α and MKK3, we treated recombinant WT p38α or the C119S p38α mutant with dBBr. Incubation of the C119S p38α mutant with dBBr resulted in a marked reduction of fluorescence emitted compared with WT p38α (Fig. 4*B*). We, however, had expected a much greater reduction, if not an absence of a fluorescence signal, because dBBr should not fluoresce in the presence of only one of the vicinal pair. Interestingly, incubation of dBBr with the double mutant also emitted a respectable fluorescence signal that was comparable with that observed with C119S. These findings suggest that the signal observed with the cysteine mutants may represent “background” fluorescence resulting from adduction by dBBr of thiols on adjacent p38α molecules that have their probability of close proximity enhanced by the high molarity of p38α used in the assay.

### Cysteine adduction attenuates the activation of p38 by MKK3

**Figure 5.**
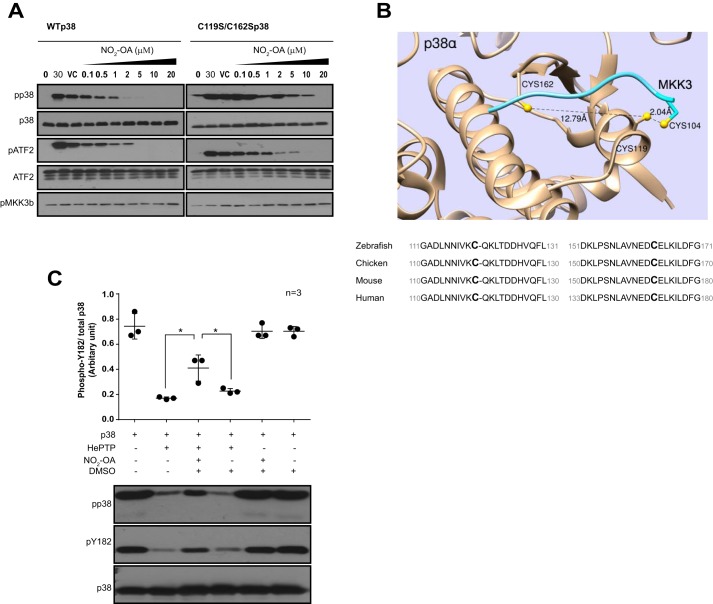
**Cysteine adduction attenuates activity of p38.** *A*, reducing SDS-PAGE and immunoblot analysis of WT p38α and C119S/C162S p38α activation by MKK3, during an *in vitro* kinase reaction in the absence or presence of 0.1–20 μm NO_2_-OA, as indicated. NO_2_-OA adduction interferes with HePTP-mediated dephosphorylation of p38α. *B*, *top*, *ribbon representation* of p38α-MKK3b in complex adapted from Protein Data Bank entry 1LEZ (7). The relevant Sulfur atoms of Cys-199 and Cys-162 within p38α and Cys-104 within MKK3b appear in *yellow* together with interatomic distances. *Bottom*, high conservation surrounding these regions of p38α. *C*, reducing SDS-PAGE and immunoblot analysis of pre-dually phosphorylated (pTGpY) p38α exposed to HePTP during an *in vitro* phosphatase reaction in the presence and absence of 25 μm NO_2_-OA. The NO_2_-OA is solubilized in DMSO. Quantification data are shown as mean ± S.D. (*error bars*). *, *p* < 0.05 *versus* NO_2_-OA, by one-way ANOVA and Newman-Keuls test.

To investigate the biochemical significance of p38-MKK3 dimerization, we examined the activity of p38α and C119S/C162S p38α by performing *in vitro* kinase assays in the presence of 10-nitro-oleic acid (NO_2_-OA), an endogenous electrophillic lipid. NO_2_-OA has been reported to post-translationally modify proteins on redox-sensitive cysteines via reversible Michael addition reactions (17). Based on the crystal structure of p38α bound to MKK3b (7), we envisaged that cysteine adduction by NO_2_-OA could potentially sterically hinder access by MKK3b, thereby preventing heterodimer formation. Dual phosphorylation of the TGY activation motif of p38α and phosphorylation of its downstream substrate, ATF-2, were used as readouts of p38 activity. In keeping with our earlier observation in cells, where the activity of p38 was not appreciably altered by the absence of the redox-sensitive cysteines (Figs. 2 (*C* and *D*) and 3*C*), both p38α and C119S/C162S p38α became phosphorylated and activated following 30-min incubation with MKK3b (Fig. 5*A*). This confirms that disabling disulfide dimer formation does not diminish MKK3 access to and consequent dual phosphorylation of p38α. Moreover, the presence of NO_2_-OA inhibited the activation of p38α but also, to a lesser extent, C119S/C162S p38α. NO_2_-OA inhibited the phosphorylation and activity of p38α at low micromolar concentrations (1 μm NO_2_-OA *versus* vehicle control), whereas a 5-fold higher concentration of NO_2_-OA (5 μm *versus* vehicle control) was required to achieve comparable inhibition of C119S/C162S p38α. The diminished sensitivity of C119S/C162S p38α suggests that NO_2_-OA selectively targets these redox-sensitive cysteines. We further interrogated this concept by examining the effect of NO_2_-OA on the activity of hematopoietic tyrosine phosphatase (HePTP), a member of a small family of phosphatases that specifically dephosphorylates p38. Like MKK3b and MEF2a, HePTP also interacts with the kinase interaction motif or the D-motif within p38, which contains one of the redox-sensitive cysteines (Cys-119), which is separated from the other (Cys-162) by 12.7 Å (Fig. 5*B*). Moreover, this cysteine pair is highly conserved (Fig. 5*B*). The incubation of dually phosphorylated (activated) p38 with HePTP for 30 min at 30 °C resulted in a marked dephosphorylation of p38 (Fig. 5*C*). This effect of HePTP was perturbed by the presence of NO_2_-OA which resulted in a moderate preservation of the dual phospho- and phospho-Tyr-182 signals in the presence of HePTP. Therefore, our experiments with NO_2_-OA indicate that it is capable of inhibiting the phosphorylation of p38 by MKK3 (Fig. 5*A*) and also its dephosphorylation by HePTP (Fig. 5*C*), most likely by adducting the redox-sensitive cysteines within p38α.

### Administration of NO_2_-OA prevents the formation of H_2_O_2_-induced dimer in murine hearts and perturbs contractile dysfunction

**Figure 6.**
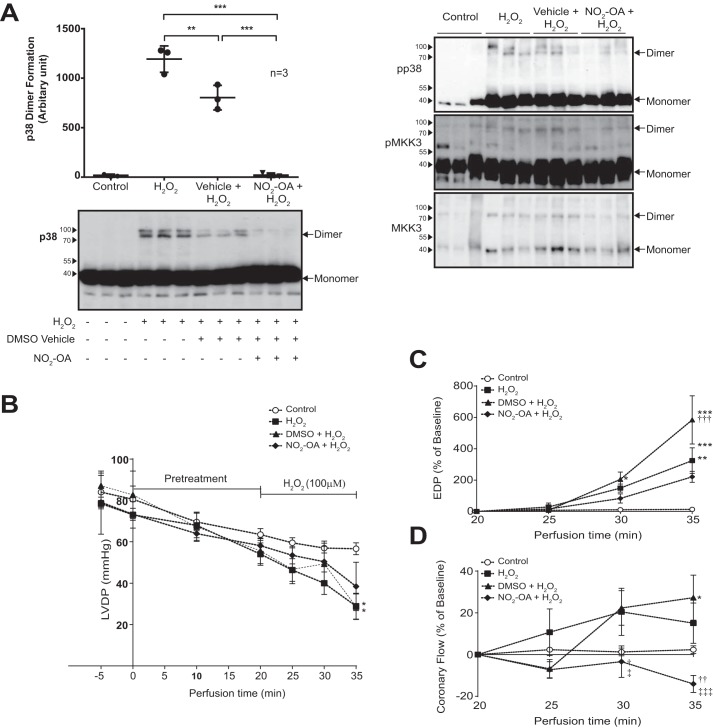
**Administration of NO_2_-OA prevents the formation of H_2_O_2_-induced dimer in murine hearts and diminishes the deleterious effect of H_2_O_2_.** *A*, non-reducing SDS-PAGE and immunoblot analysis of mouse hearts exposed to H_2_O_2_ during aerobic Langendorff perfusion, in the absence or presence of pretreatment with 10 μm NO_2_-OA in triplicate. The NO_2_-OA is solubilized in DMSO, which has an antioxidant effect and is present at 0.01% (v/v). p38 dimer quantification data are shown as mean ± S.D. from three experiments. **, *p* < 0.01; ***, *p* < 0.001 by one-way ANOVA followed by Tukey's test. *B–D*, contractile performance of mouse hearts exposed to H_2_O_2_ during aerobic Langendorff perfusion, in the absence or presence of pretreatment with 10 μm NO_2_-OA. Perfusion with 10 μm NO_2_-OA solubilized in DMSO begins at time 0 for 20 min, and perfusion with 100 μm H_2_O_2_ begins at time 20 min for 15 min. *B*, left ventricular developed pressure; *C*, end diastolic pressure; *D*, coronary flow. Data are presented as mean ± S.D. (*error bars*). The number of hearts per group was as follows: control, 6; H_2_O_2_, 6; H_2_O_2_ in the presence of DMSO, 5; and H_2_O_2_ in presence of NO_2_-OA, 5. *, *p* < 0.05 *versus* control; †, *p* < 0.05 *versus* H_2_O_2_; ‡, *p* < 0.05 *versus* H_2_O_2_ in the presence of DMSO. Comparison was by two-way ANOVA with repeated measures and post hoc comparison by Bonferroni.

To further investigate the *in vitro* observations of NO_2_-OA on p38α (Fig. 5*A*), we next examined its effect in a physiologically relevant setting. In keeping with our earlier observation in rat hearts (Fig. 1*D*), p38 disulfide dimers formed in mouse hearts following exposure to H_2_O_2_ during aerobic perfusion, an effect markedly attenuated by pre-exposure to NO_2_-OA (Fig. 6*A*). Furthermore, the administration of NO_2_-OA resulted in a reduction of contractile dysfunction mediated by H_2_O_2_ (Fig. 6, *B–D*), with no hearts excluded from the analysis. The detrimental effect of H_2_O_2_ on cardiac performance has been extensively studied and is commonly characterized by a reduction in left ventricular developed pressure, elevation in end diastolic pressure, and increased coronary flow (18–21). Accordingly, our data showed that all of these pertubations were attenuated by pretreatment with NO_2_-OA (Fig. 6, *B–D*).

### NO_2_-OA covalently modifies Cys-119 and Cys-162 of p38α

**Figure 7.**
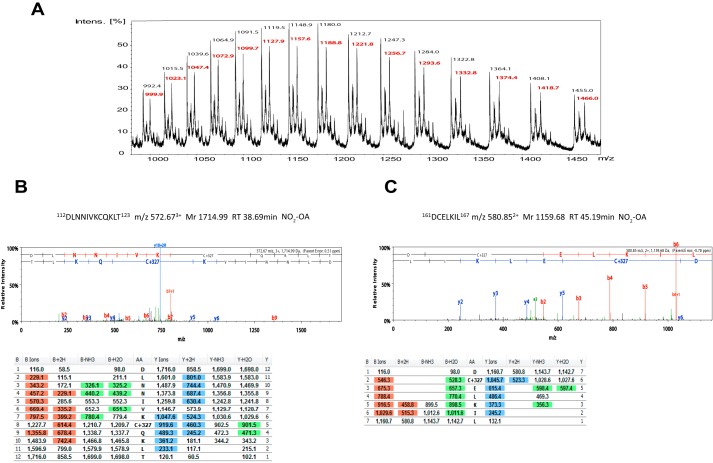
**NO_2_-OA covalently modifies the redox-sensitive cysteines of p38α**. *A*, direct infusion ESI-MS; shown in *black* is the mass of the multiple charged ion peaks belonging to non-modified p38α; shown in *boldface italic type* is the mass of the corresponding multiply charged ion peaks belonging to the NO_2_-OA–modified p38α. Multiply charged ion peaks belonging to mass/charge ratios from +30 to +44 for both species are shown. *B* and *C*, representative MS/MS spectra and fragmentation tables of NO_2_-OA–modified peptides at Cys-119 (^112^DLNNIVKCQKLT^123^) (*B*) and at Cys-162 (^161^DCELKIL^167^) (*C*). *B* shows localization of a NO_2_-OA modification on a peptide with *m*/*z* 580.85^2+^; a mass difference of 430 Da (103 + 327 Da) is detected between the y5 and y6 ions. This confirms the correct assignment of the modification to Cys-162. *C* shows localization of a NO_2_-OA modification on a peptide with *m*/*z* 572.67^3+^; a mass difference of 430 Da (103 + 327 Da) is detected between the y4 and y5 ions. This confirms the correct assignment of the modification to Cys-119. The *tables* in *B* and *C* show *highlighted* the ions of the fragmentation profile of the peptides that are detected.

We next sought to determine whether NO_2_-OA forms a covalent adduct with p38α. For this, we employed ESI-MS and LC-MS/MS analysis using two proteases to maximize sequence coverage. We identified the residues harboring nitro-fatty acid modifications, which account for the reduced phosphorylation of p38α by MKK3 (Fig. 5*A*) and reduced dephosphorylation by HePTP (Fig. 5*C*) as well as the reduced disulfide dimer formation in hearts exposed to H_2_O_2_ (Fig. 6). The spectral analysis of the ESI-MS following exposure to NO_2_-OA (Fig. 7*A*) is consistent with the formation of a covalent adduct between NO_2_-OA and p38α. Furthermore, MS/MS analysis revealed nitro-fatty acid modification of Cys-119 (Fig. 7*B*) and Cys-162 (Fig. 7*C*).

## Discussion

Here, we demonstrate that p38α has at least two redox-sensitive cysteines, 119 and 162, that lie close to a common docking domain and can influence dimerization of the key upstream activator MKK3. However, disabling disulfide dimer formation by substitution of these cysteines does not seem to influence the ability of MKK3 to dually phosphorylate p38 *in vivo* or *in vitro*. Nonetheless, when these cysteines are adducted by a biologically relevant reactive lipid, nitro-oleic acid, the binding of MKK3 and dual phosphorylation of p38α are disabled. A similar effect is seen *in vitro* with the upstream deactivator, HePTP. Our results add to the complexity of p38α signaling, suggesting that it is not merely dependent on the balance of upstream regulatory kinases and phosphatases but that an additional level of control is exercised through redox sensors within p38α.

p38 MAPK, a stress-activated kinase, is activated by divergent stimuli, including oxidative stress. It is well-known that reactive oxygen species (ROS), such as hydrogen peroxide, can induce or mediate the activation of the p38 MAPK pathway (22, 23), but the mechanisms by which ROS can activate the pathway are not well-understood. It is thought that oxidative stress is mediated by the canonical pathway of p38α activation involving TAK1, a MAP3K that lies upstream of MKK3. MKK3 is known to dock on p38α using the CD domain and then transphosphorylate the TGY motif to activate p38α. We present here data suggesting that p38α activation following exposure to H_2_O_2_ results in the formation of a disulfide bond between p38 and MKK3. This interaction has also been alluded to by others (7, 8). We observed the formation of a disulfide dimer complex containing p38 and MKK3 following exposure to H_2_O_2_ and with other oxidant insults. Although the disulfide dimer complex appeared to involve a small proportion of total p38 and MKK3 immunoreactivity, it was reversible upon exposure to a reducing agent (β-mercaptoethanol) and stabilized by auronofin, implying dynamic regulation. These observations led to the idea that the dimer was formed as a result of post-translational oxidant modification of the two proteins, p38 and MKK3. ROS are constantly generated through mitochondrial respiration and by numerous extracellular stimuli, including cytokines, growth factors, and shear stress. Under physiological conditions, they are rapidly removed by antioxidant enzymes, including superoxide dismutases, catalase, glutathione peroxidases, and peroxiredoxins, which serve to maintain a balance in the intracellular reduction–oxidation (redox) state.

One way in which ROS can alter protein structure and function is by modifying critical amino acid residues of proteins, with the thiol group of cysteine being a major target of oxidants. The oxidation of cysteine residues by H_2_O_2_ leads to formation of the disulfide bond (-S–S-) following the generation of a sulfenyl moiety (-SOH). Sulfenates are a reversible modification, and their reversibility may represent an on-off switch allowing intricate modulation of protein activity. We verified in cells overexpressing both p38α and MKK3 that the oxidant-mediated dimer formed comprised both p38α and MKK3 molecules and was not a result of homodimerization by co-expression of the epitope tags on both proteins. Moreover, immunoprecipitation of the FLAG-tagged p38α following exposure to H_2_O_2_ revealed that MKK3 indeed was complexed.

It has been previously reported that of the four cysteine residues in the human p38α primary sequence, Cys-119 and Cys-162 could potentially be involved in intermolecular disulfide bond formation because they are solvent-exposed and located in flexible surface loops (24). Cys-39 and Cys-211 are buried in the molecular interior and thus not likely to be involved in oxidation phenomena. Cys-119 and Cys-162 are highly conserved in zebrafish, mouse, human, etc. and could be involved in redox regulation of p38 (Fig. 5*B*). As organisms have increased in complexity, the cysteine content of their constituent proteins has increased from 0.41% in Archaea to 2.26% in mammals (25). A small component of this expansion seems to relate to zinc-binding proteins or enzymes with catalytic thiols, an observation suggesting that the majority of evolutionary change relates to the acquisition of redox sensitivity. This is in keeping with our findings because Cys-119 and Cys-162 show strong evolutionary conservation (Fig. 5*B*). Cys-162, among many other residues, was also recently reported to be electrophilic in a large quantitative MS-based screen developed for the identification of cysteine persulfides in mammalian cells (26). Cys-109 within MKK6 was previously reported to be involved in formation of an intramolecular disulfide bond upon oxidation that inactivates MKK6 by inhibiting its ATP binding (27). Consistent with these findings, Cys-104 is the corresponding residue within MKK3 that reputedly oxidizes and forms the disulfide bond with p38α in our study. Furthermore, the authors also described a pair of cysteines that could form an intramolecular disulfide bond, much akin to our observations that Cys-119 and Cys-162 acting as vicinol thiols. Collectively, these observations substantiate Cys-104 within MKK3 as having redox potential and the notion of intra- and intermolecular disulfide bonds being involved in MAP2K and MAPK activation as conceivable. The formation of the dimer was dependent on redox-sensitive cysteines, because the interaction between p38 and MKK3 was abrogated when the cysteines had been mutated to non-oxidizable serine residues. We further exploited this notion by impeding the oxidant-mediated interaction between MKK3 and p38 using the electrophilic lipid, NO_2_-OA, which resulted in a reduction in p38α activity, which also diminished in the absence of the redox-sensitive cysteines within C119S/C162S p38.

NO_2_-OA, which is a component of the Mediterranean diet, has received attention due to its protective effects in murine models of hypertension (17, 28), cardiac (29), and renal (30) ischemia–reperfusion injury. Based on the crystal structure of p38α, we hypothesized that NO_2_-OA-cysteine adduction via reversible Michael addition reactions (17) on either cysteine residue should cause steric hindrance and prevent formation of a dimer with MKK3 (see Fig. 5*A*). To be sure that NO_2_-OA could directly interact with p38α, we conducted LC-MS/MS analysis and confirmed that NO_2_-OA indeed covalently adducted p38α, specifically upon Cys-119 and Cys-162, as predicted. Our observations on the effect of NO_2_-OA on modulation of the p38 D-motif-interacting HePTP phosphatase and the exposure of Langendorff-perfused hearts to NO_2_-OA prior to oxidant stress by H_2_O_2_, which resulted in a significant attenuation of p38 disulfide dimer formation and also the alleviation of H_2_O_2_-mediated contractile dysfunction, together complement our characterization of a novel mode of p38 regulation, not described elsewhere thus far. These observations may in part explain the anti-inflammatory effects of NO_2_-OA (31, 32).

The D-motif–binding site is responsible for conferring selectivity to the binding partners of p38; these include activators (MKK3), substrates (TAB1, MEF2C), and others (phosphatases). The docking strategy enables p38 to be selectively regulated by diverse signals, which must in turn also translate to specific outcomes. Our results suggest that an additional level of control is exerted by redox modification of cysteines adjacent to this binding motif that sterically control access to p38. However, although substitution of these residues abolished p38-MKK3 disulfide dimer formation, this did not seem to impact on p38 activation.

## Experimental procedures

### Adult rat ventricular myocyte culture

ARVMs were isolated from adult male Wistar rats by collagenase-based enzymatic digestion as described previously (33) and washed with M199 complete medium (M199 medium with added 100 IU/ml penicillin, 100 IU/ml streptomycin, 2 mm l-carnitine, 5 mm creatine, and 5 mm taurine). The cell suspension was allowed to settle by gravity and then resuspended in M199 complete medium and placed in laminin-coated 6-well plates before incubation in 5% CO_2_, room air at 37 °C. After 1 h, the medium was aspirated, leaving only adherent cells, and fresh prewarmed M199 complete medium was added. Following overnight culture, cells were exposed to the oxidant stresses, H_2_O_2_ (100 and 500 μm for 10 min), peroxynitrite (500 μm for 30 or 60 min), SIN-1 (200 μm for 5, 15, 30, 60, or 90 min), or diamide (50 μm for 10 min). Cells were harvested in 2× SDS sample buffer containing 100 μm maleimide.

### Retrograde perfusion of isolated rodent hearts

All procedures were performed in accordance with the United Kingdom Home Office Guidance on the Operation of the Animals (Scientific Procedures) Act 1986.

Male Wistar rats (220–250 g) were anesthetized by intraperitoneal injection with sodium pentobarbital (200 mg/kg) and heparin (200 IU/kg). Hearts were rapidly excised and placed in ice-cold modified Krebs-Henseleit buffer (KHB) (118.5 mm NaCl, 25.0 mm NaHCO_3_, 4.75 mm KCl, 1.18 mm KH_2_PO_4_, 1.19 mm MgSO_4_, 11.0 mm d-glucose, and 1.4 mm CaCl_2_, pH 7.4). The excised hearts were cannulated via the aorta and perfused with oxygenated (95% O_2_ and 5% CO_2_) KHB at 37.0 °C in a retrograde Langendorff perfusion system at a constant pressure equivalent to 72 ± 1 mm Hg using a peristaltic pump and a feedback system controlled by an STH pump controller (AD Instruments, Oxford, UK). More detailed methods are described elsewhere (34). Following a 25-min stabilization, the hearts were perfused with KHB with H_2_O_2_ (50, 100, 200, and 500 μm) for 15 min.

The effect of NO_2_-OA on the formation of p38 dimer in hearts exposed to H_2_O_2_ was examined. C57BL/6 mice (22–28 g; Harlan) were anesthetized by intraperitoneal pentobarbital (300 mg/kg) and heparin (150 IU/kg). Hearts were rapidly excised and placed in ice-cold modified KHB. The hearts were perfused at a constant pressure of 80 mm Hg with KHB equilibrated with 95% O_2_ and 5% CO_2_ at 37 °C. Atrial pacing was performed ∼600 beats/min. Hearts were stabilized for 30 min after the initiation of retrograde perfusion. For inclusion, all hearts had to fulfill the following criteria: time from thoracotomy to aortic cannulation < 3 min, coronary flow between 1.5 and 4 ml/min, and no persistent dysrhythmias during the stabilization period. The hearts were exposed to 10 μm NO_2_-OA or vehicle control (DMSO) for 30 min prior to administration of 100 μm H_2_O_2_ for 10 min. Hearts were rapidly snap-frozen. Hearts were thawed and homogenized in extraction buffer (50 mm Tris-HCl, pH 7.5, 1 mm EDTA, 1 mm EGTA, 1 mm Na_3_VO_4_, 50 mm NaF, 5 mm sodium pyrophosphate, 1% Triton, and 1 cOmplete® protease inhibitor tablet (Roche Diagnostics)). Heart homogenates were centrifuged at 4 °C for 10 min at 13,000 r.p.m., and supernatant was resuspended in 2× SDS buffer containing 100 μm maleimide, boiled for 10 min, and resolved by SDS-PAGE. In a separate set of experiments, the effect of NO_2_-OA on the contractility of hearts exposed to H_2_O_2_ was assessed. An intraventricular fluid-filled compliant balloon attached to a pressure transducer coupled to a 4S Powerlab system (AD Instruments) allowed the continuous monitoring of contractile function, left ventricular systolic and diastolic pressures and (by difference) left ventricular developed pressure and coronary flow. Hearts were exposed to 10 μm NO_2_-OA or vehicle control (DMSO) for 20 min prior to administration of 100 μm H_2_O_2_ for 15 min.

### Cell culture and transfection

HEK293 and H9C2 cell lines (ATCC) were maintained in DMEM supplemented with 10% FCS and 100 IU/ml penicillin and 100 IU/ml streptomycin in 5% CO_2_, room air at 37 °C. Cells were cultured in 6-well plates and transfected at 70% confluence in Opti-MEM (Life Technologies) using Turbofect^TM^ reagent (Thermo Scientific) and the relevant plasmid DNAs. Wild-type p38α MAPK-expressing plasmid was obtained from Jiahuai Han (Scripps Research Institute, La Jolla, CA) (35). p38α constructs with either Cys-119 or Cys-162 or both mutated to serine were generated by QuikChange® site-directed mutagenesis (Stratagene). MKK3 was from Par Gerwins (University of Uppsala, Uppsala, Sweden) (36). All p38α constructs were FLAG-tagged, and MKK3 was hemagglutinin-tagged and cloned into the mammalian expression plasmid pcDNA3 (Invitrogen). 24 h post-transfection, cells were exposed to 100 μm H_2_O_2_ for 10 min with or without pre-exposure to 10-nitro-oleic acid and harvested in 2× SDS sample buffer containing 100 μm maleimide.

### Co-immunoprecipitation

Cells were washed in ice-cold PBS and lysed in 400 μl of iced lysis buffer (50 mm Tris-HCl, pH 7.4, 150 mm NaCl, mm EDTA, 1% Triton X-100, one protease inhibitor mixture tablet per 50 ml (cOmplete®, Roche Diagnostics)). The cells were scraped and collected into cooled microcentrifuge tubes. Cell lysates were centrifuged at 12,000 × *g* for 10 min at 4 °C. Supernatants were transferred into chilled tubes containing 40 μl of a 50% anti-FLAG M2 affinity gel slurry (Sigma), prepared as per the manufacturer's instructions. Following overnight incubation on a rotation wheel, at 4 °C, samples were centrifuged at 6,000 × *g* for 30 s to pellet the anti-FLAG M2 affinity gel-bound proteins, followed by three washes with 0.5 ml of lysis buffer. Samples were resuspended in 2× SDS sample buffer containing 100 μm maleimide.

### Immunoblot analysis

Samples were resolved on a 10% SDS-polyacrylamide gel under non-reducing conditions and transferred onto PVDF membranes. After blocking in 4% nonfat milk and 1% BSA in Tris-buffered saline, pH 7.4, for 1 h, membranes were exposed to the following primary antibodies: anti-dual phospho-p38 MAPK (Thr-180 and Tyr-182; 9211, Cell Signaling), anti-p38 MAPK (9212, Cell Signaling), anti-phospho MEK3 (ab131283, Abcam), MEK3 (ab47522, Abcam), anti-phospho ATF-2 (9221, Cell Signaling), anti-ATF-2 (9226, Cell Signaling), anti-HA (2367, Cell Signaling), or anti-FLAG (2368, Cell Signaling) all at 1:1,000 overnight at 4 °C with agitation. After washing and incubation with HRP-conjugated secondary antibody (1:2,000) (NA934V, GE Healthcare), antigen–antibody complexes were visualized by enhanced chemiluminescence detection (Pierce).

### Expression and purification of WT p38α, p38α C119S, p38α C162S, and p38α C119S/C162S recombinant proteins

DNA encoding p38α was derived from a pET14b vector kindly donated by Y. Wang (37) and subcloned into a pETDuet-1 vector. The full coding sequence included an N-terminal His_6_ tag followed by a tobacco etch virus cleavage site and mouse p38α. The p38α mutants were generated by overlapping C and N terminus fragments produced by PCR with complementary internal primers containing the desired mutations. The PCR products were then combined to form the template for a second PCR with external primers. The vectors were transformed in *E. coli* strain Rosetta II cells (Novagen). The protein expression and purification procedure followed was as described previously (38). Following dialysis overnight at 4 °C in 100 mm NaCl, 20 mm Tris, and 5 mm TCEP (Invitrogen) (pH 7.5), purified proteins were aliquoted and stored at −80 °C.

### Vicinal thiol analysis of p38α

The formation of an intramolecular disulfide bond between Cys-119 and Cys-162 in p38α was measured using dBBr. dBBr is a fluorogenic homobifunctional thiol-specific cross-linking reagent that is non-fluorescent until both of its alkylating groups have been reacted by vicinal thiols. It then emits light, which can be measured at 477 nm.

75 μg of recombinant wild-type p38α (WT p38α) was transferred into a cooled tube containing 100 mm Tris buffer (pH 7.4) and reduced by the addition of TCEP (Invitrogen) to a final concentration of 5 mm for 15 min at room temperature. The TCEP concentration was further diluted to 50 μm by the addition of 100 mm Tris buffer (pH 7.4). 2.5 μg (w/v) of the reduced protein was then transferred to a well of a black 96-well plate (Sterilin, ThermoFisher Scientific) containing either 100 mm Tris buffer (pH 7.4) alone or with 500 μm H_2_O_2_ or 1 mm diamide and incubated for 15 min at room temperature. Finally, 100 μm dBBr (Invitrogen) was added, and samples were incubated at room temperature for 15 min in the dark before monitoring fluorescence in a plate reader with fluorescence excitation/emission spectra of 385/477 nm.

TCEP was removed from 75 μg of recombinant WT p38α, C119S p38α, or C119S/C162S p38α using 7,000 molecular weight cut-off Zeba^TM^ spin desalting columns (Thermo Scientific) as per the manufacturer's instructions. 2.5 μg (w/v) of the desalted protein sample was immediately transferred to the wells of a 96-well plate containing 100 mm Tris buffer (pH 7.4) and 100 μm dBBr and incubated at room temperature for 15 min in the dark before monitoring fluorescence in a plate reader with fluorescence excitation/emission spectra of 385/477 nm.

### Simulated ischemia/reperfusion protocol

Simulated ischemia was induced by exposing H9c2 cells cultured on 6-well plates for the specified duration to modified KHB (137 mm NaCl, 3.58 mm KCl, 0.49 mm MgCl_2_, 1.8 mm CaCl_2_, and 4.0 mm HEPES) with 20 mm 2-deoxyglucose, 20 mm sodium lactate, and 1 mm sodium dithionite at pH 6.5 in 5% CO_2_, room air at 37 °C (13). The buffer was aspirated, and then cells were either collected in 2× SDS sample buffer containing 100 μm maleimide or reperfused by the addition of serum-free DMEM for the specified duration before collecting in 2× SDS sample buffer containing 100 μm maleimide.

### In vitro kinase assay

The effect of the endogenous electrophilic lipid, NO_2_-OA, on the activation and activity of p38 *in vitro* was examined. Purified recombinant p38α (0.1 μm) was incubated with NO_2_-OA (10037, Cayman Chemical) in buffer containing 100 mm NaCl, 20 mm Tris, pH 7.5, 2 mm MgCl, and 200 μm ATP at 37 °C for 30 min. The reaction, in a 50-μl final volume, was started by the addition of 0.1 μg of MKK3b, 0.5 μg of ATF-2 fusion protein (9224, Cell Signaling) and incubated at 30 °C for 30 min. The reaction was stopped by the addition of 5× SDS sample buffer, and samples were resolved by SDS-PAGE and transferred onto PVDF membranes.

The effect of NO_2_-OA on the ability of HePTP to dephosphorylate p38α was examined. Purified recombinant activated dually phosphorylated p38α (3 μm) was prepared as described previously (34) and incubated with NO_2_-OA (15 μm) in buffer containing 5 mm MOPS, 10 mm NaCl, 10 μmm EDTA, pH 7.0, at 37 °C for 30 min. The reaction, in a 40-μl final volume, was started by the addition of 0.3 μm purified recombinant HePTP, prepared and purified from a plasmid vector kindly donated by W. Peti (39) and incubated at 30 °C for 30 min. The reaction was stopped by the addition of 5× SDS sample buffer, and samples were resolved by SDS-PAGE.

### Liquid chromatography tandem mass spectrometry (LC-MS/MS) analysis

ESI-MS and LC-MS/MS were carried out to determine whether NO_2_-OA forms a covalent adduct with p38α. p38α at 1 mg/ml was treated with equimolar 10 nitro-oleic acid at room temperature for 1 h. The reaction mixture was then analyzed by direct-infusion ESI-MS and LC-MS/MS. To improve sequence coverage with LC-MS/MS, both trypsin and Asp-N were used to digest p38α.

For enzymatic digestion, in-solution reduction, alkylation, and digestion with trypsin or Asp-N of total p38α protein was performed prior to subsequent analysis by mass spectrometry by following methods described previously (40). Briefly, cysteine residues were reduced with TCEP, because the NO_2_-OA modification adduct is not stable in the presence of dithiothreitol, and derivatized by treatment with iodoacetamide to form stable carbamidomethyl derivatives. Trypsin and Asp-N digestion was carried out for 16 h at 37 °C.

### LC-MS/MS

Chromatographic separations were performed using an EASY NanoLC system (ThermoFisher Scientific). Peptides from a total protein amount of 2 mg on column were resolved by reversed phase chromatography on a 75-μm C18 column using a three-step linear gradient of acetonitrile in 0.1% formic acid. The gradient was delivered to elute peptides at a flow rate of 300 nl/min over 60 min. The eluate was ionized by electrospray ionization using an Orbitrap Velos Pro (ThermoFisher Scientific), operating under Xcalibur version 2.2. The instrument was programmed to acquire in automated data-dependent switching mode, selecting precursor ions based on their intensity for sequencing by collision-induced fragmentation using a Top20 collision-induced dissociation method. The MS/MS analyses were conducted using collision energy profiles that were chosen based on the mass-to-charge ratio (*m*/*z*) and the charge state of the peptide.

## Author contributions

M. S. M. conceived and coordinated the study and wrote the paper. R. B. wrote the paper and designed, performed, and analyzed the experiments shown in Figs. 1–6. J. R. B. and P. E. provided intellectual and technical assistance throughout and contributed to final revisions of the manuscript. G. F. D. designed, performed, and analyzed the experiments shown in Fig. 7. O. R. performed the experiments shown in Fig. 1*D*. V. D. performed the experiment shown in Fig. 5*C*. R. L. C. provided technical assistance with the design of the experiments shown in Fig. 4. All authors reviewed the final version of the manuscript.

## Footnotes

2 The abbreviations used are:

AVRM: adult rat ventricular myocyte
SI: simulated ischemia
dBBr: dibromobimane
HePTP: hematopoietic tyrosine phosphatase
ROS: reactive oxygen species
TCEP: tris(2-carboxyethyl)phosphine
ANOVA: analysis of variance
KHB: Krebs-Henseleit buffer
NO_2_-OA: 10-nitro-oleic acid.
